# Obesity classification predicts early complications and mortality after acetabular fracture

**DOI:** 10.1007/s00590-023-03633-8

**Published:** 2023-07-06

**Authors:** Julian Wier, Reza Firoozabadi, Joseph T. Patterson

**Affiliations:** 1 Department of Orthopaedic Surgery, Keck School of Medicine of the University of Southern California, 1520 San Pablo Street, Suite 2000, Los Angeles, CA 90033-5322 USA; 2 Department of Orthopedics and Sports Medicine, University of Washington, Seattle, WA USA

**Keywords:** Obesity, Acetabular fracture, Complications, Operative, Non-operative, Inpatient, Body mass index

## Abstract

**Introduction:**

Obesity remains a global epidemic. The effect of obesity on the risk of complications after acetabular fracture is unknown. Here, we evaluate the effect of BMI on early complications and mortality after acetabular fracture. We hypothesize that the risk of inpatient complications and mortality will be greater in patients with high BMI when compared to those with normal BMI.

**Methods:**

Adult patients with acetabular fracture were identified via the Trauma Quality Improvement Program data from 2015 to 2019. The primary outcome was overall complication rate with reference to normal-weight patients (BMI = 25–30 kg/m^2^). The secondary outcome was rates of death. The association of obesity class on the primary and secondary outcomes was assessed using Bonferroni-corrected multiple logistic regression models considering patient, injury, and treatment covariates.

**Results:**

A total of 99,721 patients with acetabular fracture were identified. Class I obesity (BMI = 30–35 kg/m^2^) was associated with 1.2 greater adjusted relative risk (aRR; 95% confidence interval (CI) 1.1–1.3) of any adverse event, without significant increases in adjusted risk of death. Class II obesity (BMI = 35–40 kg/m^2^) was associated with aRR = 1.2 (95% CI 1.1–1.3) of any adverse event and aRR = 1.5 (95% CI 1.2–2.0) of death. Class III obesity (BMI ≥ 40 kg/m^2^) was associated with aRR = 1.3 (95% CI 1.2–1.4) of any adverse event and aRR = 2.3 (95% CI 1.8–2.9) of death.

**Conclusion:**

Obesity is associated greater risk of adverse events and death following acetabular fracture. Obesity severity classification scales with these risks.

**Supplementary Information:**

The online version contains supplementary material available at 10.1007/s00590-023-03633-8.

## Introduction

Obesity is a preventable disease and an emerging global pandemic [1]. Obesity currently accounts for the greatest number of disability-adjusted life years lost in adults and is anticipated to become the most fatal metabolic disease among adults by 2050 [2]. In the US, 42% of adults in the US, 17% in the European Union, and 13% worldwide meet the World Health Organization (WHO) definition of obesity of a body mass index (BMI) greater than 30 kg/m^2^ [3].

Obesity is associated with increased morbidity after trauma as well as higher rates of perioperative complications and longer hospital stays [4, 5]. Obesity is also a risk factor for common complications of acetabular fracture, including deep-vein thrombosis, respiratory failure, and infection [5–9]. However, there is conflicting evidence on whether obesity independently modifies the risk of complications after an acetabular or pelvic fracture [10, 11]. An “obesity paradox” has been described in which the risk of complications appears lower in obese patients than in normal-weight patients following trauma [12].

We investigate whether the WHO classes of obesity are independently associated with the risk of early complications following admission for trauma involving an acetabular fracture. To our knowledge, no sufficiently powered prior studies have examined the granularity of this relationship. We hypothesize that the risk of early inpatient complications is associated with the severity of obesity classification.

## Patients and methods

We used the Trauma Quality Improvement Program (TQIP) data from the American College of Surgeons to retrospectively identify adult patients (≥ 18 years) who presented to trauma centers between 2015 and 2019 with a new diagnosis of acetabular fracture. TQIP is a deidentified database of injured patients presenting to over 875 North American trauma centers [13]. Abbreviated Injury Scale (AIS 2005) codes were used to identify acetabular fractures (Supplementary Table S1) using National Trauma Data Standard (NTDS) criteria. Use of open reduction internal fixation (ORIF) or percutaneous/closed reduction internal fixation (CRIF) for acetabular fractures was identified via International Statistical Classification of Diseases and Related Health Problems 10th coding, Procedural Coding System codes (ICD-10-PCS; Supplementary Table S1). Patients were excluded if declared dead on arrival, had disseminated cancer, or current chemotherapy use. Covariates with > 20% missing data for primary study outcomes or model covariables were excluded.

The multi-level ordinal exposure of the WHO obesity class was categorized using BMI [3]. The primary outcome was any inpatient complication, defined as at least one instance of acute kidney injury (AKI), cardiac arrest, central line-associated bloodstream infection (CLABSI), catheter-associated urinary tract infection (CAUTI), deep surgical site infection (SSI), deep-vein thrombosis (DVT), intubation, osteomyelitis, pulmonary embolism (PE), pressure ulcer, respiratory failure, sepsis, stroke, superficial SSI, unplanned admission to the intensive care unit (ICU), unplanned return to the operating room (OR), or ventilation-associated pneumonia (VAP). Secondary outcomes included serious adverse events (SAE), infectious complications, and death. SAEs were defined as AKI, death, deep SSI, DVT, intubation, cardiac arrest, PE, respiratory failure, sepsis, stroke, unplanned ICU admission, unplanned return to the OR, or VAP. Infectious complications were defined as CAUTI, CLABSI, superficial or deep SSI, osteomyelitis, sepsis, organ space infection, or VAP.

Patient demographic data, medical history, injury characteristics, vital signs within the first hour of emergency department/hospital presentation, and initial treatments putatively associated with obesity or any patient complication were collected (Supplementary Table S2). Confounding variables were defined as those variables from this set that, when included in the multivariable logistic regression model, resulted in a > 10% change in the β estimate. The grouped smooth and fractional polynomial approaches were used to evaluate the linearity assumption of logistic regression. Competing models were assessed using the likelihood ratio test, and model fit was evaluated via inspection of residuals and outliers. Multicollinearity was assessed via evaluation of variance inflation factors (VIF). Covariates with VIF > 10 were excluded from the model in a stepwise fashion. These analyses were repeated for SAEs, infectious complications, and death. To better understand the subtype of systemic or local complications, a further multivariable analysis was conducted for the risk of acute kidney injury, cardiac complication (MI or cardiac arrest), pulmonary complication (respiratory failure, unplanned intubation, or VAP), SSI (deep or superficial in patients treated with ORIF or CRIF only), and venous thromboembolism (VTE).

All statistical analyses were performed using Stata Version 17.0 (College, Station, TX), reporting two-sided *p* values with the level of significance of *p* < 0.050. Significance for the study outcomes was set at *p* < 0.0125 after implementing a Bonferroni correction for multiple hypothesis testing.

## Results

**Fig. 1:**
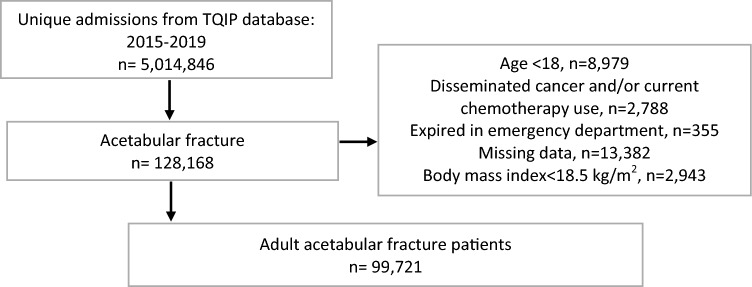
STROBE diagram of patient selection

A total of 99,721 patients with acetabular fractures were eligible for this analysis (Fig. 1). The mean age was 49.0 years (95% confidence interval (CI) 48.9–49.1 years), and the majority were male (66.5%) and White (68.0%) (Table 1). The mean BMI was 28.8 kg/m^2^ (95% CI 28.7–28.8 kg/m^2^), with the majority of patients either normal weight (33.5%) or overweight (33.4%). Obesity classes I–III composed 18.2%, 7.9%, and 7.0% of the cohort, respectively. Patients presented with an average ISS of 13.9 (95% CI 13.8–13.9) and with largely moderate to serious injuries by body region (Table 1). Concomitant pelvic ring disruptions (55.0%), femoral (17.2%), and tibial (11.2%) fractures were common. One-column acetabular fractures comprised 47.1% of cases, while transverse (10.3%) and associated both column (12.7%) fractures were less frequent. About 3.9% of acetabular fractures were open. About 25.5% of patients received ORIF for their acetabular fracture, 1.9% received CRIF, and 72.7% received non-operative treatment. Acetabular fracture repair typically occurred 1–3 days (43.5%) after admission. Most admissions for acetabular fracture occurred at large academic level 1 trauma centers (Table 2). Further treatment data are reported in Table 2.

**Table 1:** Demographic and injury characteristics

	*N*	Mean (95% CI)/%
Age, years		49.0 (48.9, 49.1)
Sex, male	66,902	67.1%
Race		
American Indian, yes	738	0.7%
Asian, yes	1715	1.7%
Black, yes	14,773	14.8%
Hispanic, yes	14,523	14.6%
Pacific Islander, yes	268	0.3%
Other, yes	22	0.0%
White, yes	67,682	67.9%
BMI kg/m^2^		28.8 (28.7, 28.8)
Normal weight, yes	33,404	33.5%
Overweight, yes	33,279	33.4%
Class I obesity, yes	18,107	18.2%
Class II obesity, yes	7903	7.9%
Class III obesity, yes	6984	7.0%
*Comorbidities*		
Alcoholism, yes	5804	5.8%
Angina, yes	996	1.0%
Anticoagulant use, yes	3411	3.4%
Bleeding disorder, yes	3109	3.1%
CHF, yes	2927	2.9%
Cirrhosis, yes	1566	1.6%
COPD, yes	5193	5.2%
CVA history, yes	2312	2.3%
Dementia, yes	2911	2.9%
Diabetes, yes	12,030	12.0%
ESRD, yes	1917	1.9%
Functional dependence, yes	3953	4.0%
Hypertension, yes	22,854	23.9%
Mental disorder, yes	8552	8.6%
Myocardial infarction history, yes	1445	1.5%
PAD, yes	1304	1.3%
Smoking, yes	21,220	21.3%
Steroid use, yes	1500	1.5%
Substance abuse, yes	7697	7.7%
*Vitals and injury characteristics*		
SBP, mmHg		120.9 (120.7, 121.1)
Pulse rate		94.8 (94.6, 95.0)
Respiratory rate		19.9 (19.9, 20.0)
GCS		13.9 (13.9, 13.9)
ISS		13.9 (13.8, 13.9)
AIS Region 1, Head		2.4 (2.4, 2.4)
AIS Region 2, Face		1.3 (1.3, 1.3)
AIS Region 3, Neck		1.9 (1.9, 2.0)
AIS Region 4, Thorax		2.7 (2.7, 2.7)
AIS Region 5, Abdomen		2.3 (2.3, 2.3)
AIS Region 6, Spine		2.2 (2.2, 2.2)
AIS Region 7, Upper extremity		1.7 (1.7, 1.7)
AIS Region 8, Lower extremity		2.5 (2.4, 2.5)
*Acetabular fracture type*		
One column, yes	47,009	47.1%
Transverse, yes	10,241	10.3%
Associated both columns, yes	12,636	12.7%
Other, yes	29,835	29.9%
Open, yes	3917	3.9%
Concomitant femur fracture, yes	17,185	17.2%
Concomitant tibia fracture, yes	11,120	11.2%
Concomitant pelvic ring disruption, yes	54,831	55.0%

AIS, Abbreviated Injury Score; BMI, Body Mass Index; CHF, Congestive Heart Failure; CI, Confidence Interval; COPD, Chronic Obstructive Pulmonary Disease; CVA, Cerebrovascular Accident; ESRD, End-Stage Renal Disease; GCS, Glasgow Coma Scale; ISS, Injury Severity Score; PAD, Peripheral Artery Disease; and SBP, Systolic Blood Pressure

**Table 2:** Admitting hospital characteristics and interventions employed

	*N*	Mean/%
*Trauma level*		
I, yes	50,859	51.0%
II, yes	27,490	27.6%
III, yes	2982	3.0%
N/A, yes	18,390	18.4%
*Hospital bed size*		
< 200, yes	6123	6.1%
200–400, yes	25,644	25.7%
401–600, yes	28,167	28.3%
> 600, yes	39,787	39.9%
Teaching status, yes	50,349	50.6%
Interfacility transfer, yes	29,431	29.5%
Time in ED, minutes		242.5 (226.9, 258.1)
*Definitive acetabular fracture fixation*		
Non-operative, yes	72,460	72.7%
CRIF, yes	1871	1.9%
ORIF, yes	25,390	25.5%
*Time to acetabular fixation*		
< 24 h	6166	22.9%
1–3 days	11,719	43.5%
3.1–5 days	5356	19.9%
> 5 days	3719	13.8%
Exploratory laparotomy, yes	3309	3.3%
Angioembolization of pelvic vessels, yes	1278	1.3%
*Units of pRBCs given within 24 h of admission*		
None, yes	88,467	88.8%
< 1 unit, yes	380	0.4%
1–4 units, yes	4811	4.8%
4.1–8 units, yes	3113	3.1%
8.1–12 units, yes	1360	1.4%
> 12 units, yes	1590	1.6%
*VTE prophylaxis type*		
Direct thrombin inhibitor, yes	72	0.1%
Factor Xa inhibitor, yes	642	0.6%
Heparin, yes	8940	9.0%
LMWH, yes	66,979	67.2%
Warfarin, yes	403	0.4%
Other, yes	3392	3.4%
None, yes	2329	2.3%
Unknown, yes	16,964	17.0%

CRIF, Closed Reduction Internal Fixation; ED, Emergency Department; LMWH, Low-Molecular-Weight Heparin; LOS, Length of Stay; ORIF, Open Reduction Internal Fixation; pRBC, Packed Red Blood Cells; and VTE, Venous Thromboembolism

At least one inpatient complication occurred in 36.5% of patients. SAE and infectious complications were observed in 35.6% and 11.6% of patients, respectively. In-hospital mortality was 3.4%. The median length of stay was 7 days (interquartile range (IQR) 4–12 days). The 38.8% of patients requiring ICU admission remained in the unit for a median of 4 days (IQR 3–9 days). About 19.7% of patients required mechanical ventilation for a median of 4 days (IQR 2–10 days).

The unadjusted rates of any inpatient complication, SAE, infection, and death were associated with higher obesity classes regardless of acetabular fracture management; increasing total hospital days, ICU days, and ventilator days were associated with increasing BMI (Supplementary Table S3).

**Fig. 2:**
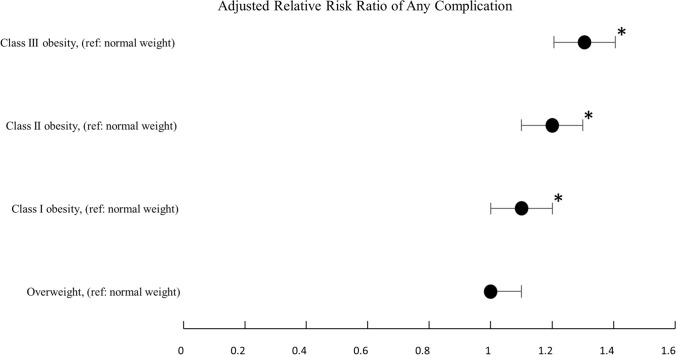
Adjusted relative risk ratio of any inpatient complication compared to normal weight by body mass index class (*denotes *p* < 0.0125)

Multiple logistic regression models demonstrated that the WHO obesity class was significantly associated with increased adjusted relative risk (aRR) of any inpatient complication with reference to normal-weight patients: in class I obesity aRR = 1.2 (95% CI 1.1–1.3, *p* = 0.012), class II obesity aRR = 1.2 (95% CI 1.1–1.3, *p* < 0.001), and class III obesity aRR 1.3 (95% CI 1.2–1.4, *p* < 0.001) (Fig. 2; Supplementary Table S4).

**Fig. 3:**
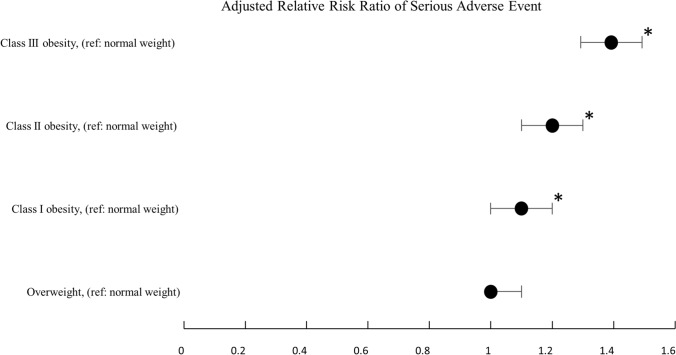
Adjusted relative risk ratio of serious adverse event compared to normal weight by body mass index class (*denotes *p* < 0.0125)

The risk of serious adverse events was significantly associated with the WHO obesity class: class I obesity aRR = 1.1 (95% CI 1.1–1.2, *p* = 0.005), class II obesity aRR = 1.2 (95% CI 1.1–1.3, *p* < 0.001), and class III obesity aRR 1.4 (95% CI 1.3–1.5, *p* < 0.001) when compared to normal-weight patients (Fig. 3; Supplementary Table S5).

**Fig. 4:**
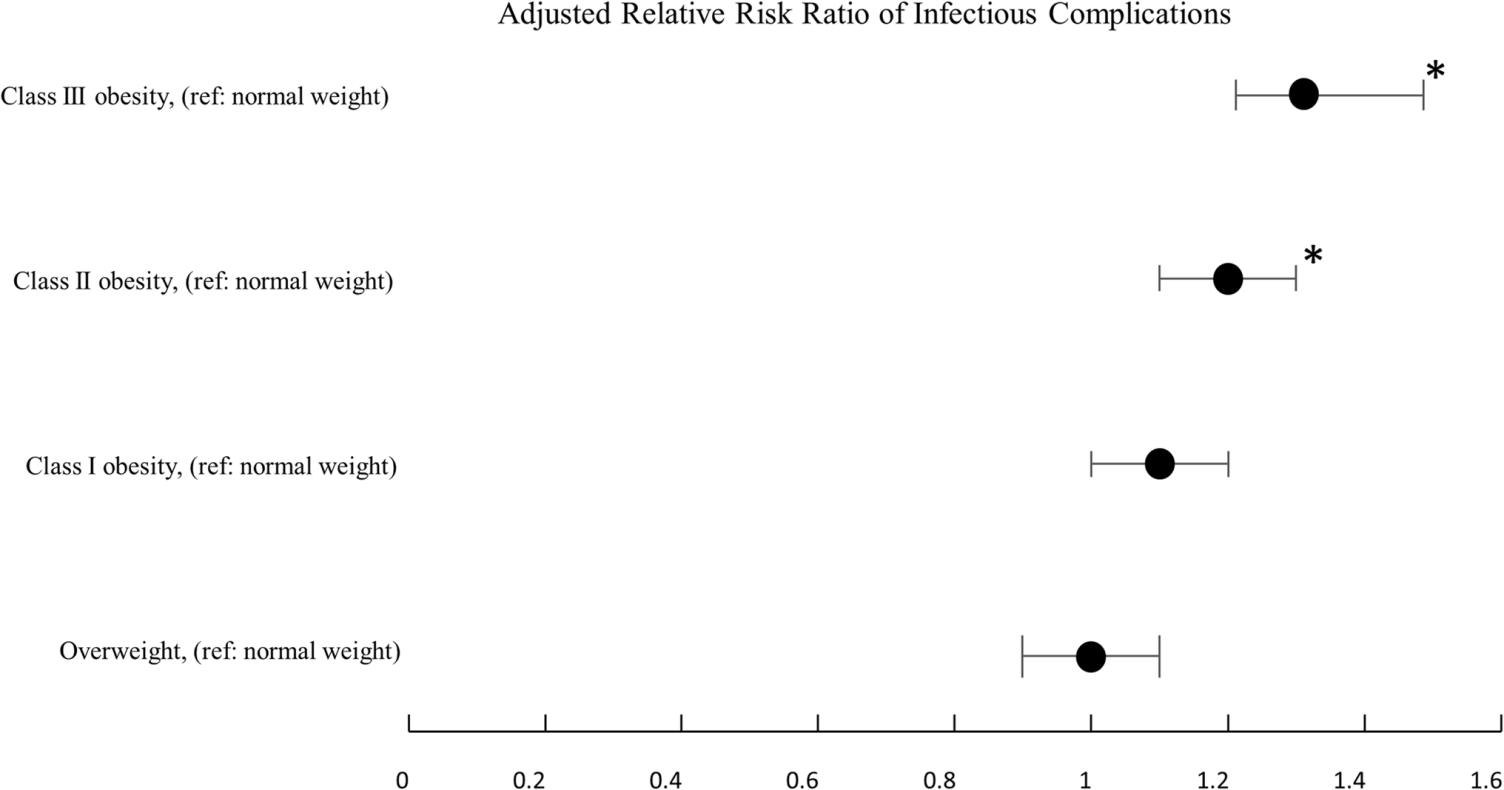
Adjusted relative risk ratio of infectious complications compared to normal weight by body mass index class (*denotes *p* < 0.0125)

The risk of infectious complications was significantly associated with the WHO obesity class: class II obesity aRR = 1.2 (95% CI 1.1–1.3, *p* = 0.005) and class III obesity (aRR = 1.3 (95% CI 1.2–1.5, *p* < 0.001) patients when compared to normal-weight patients (Fig. 4; Supplementary Table S6).

**Fig. 5:**
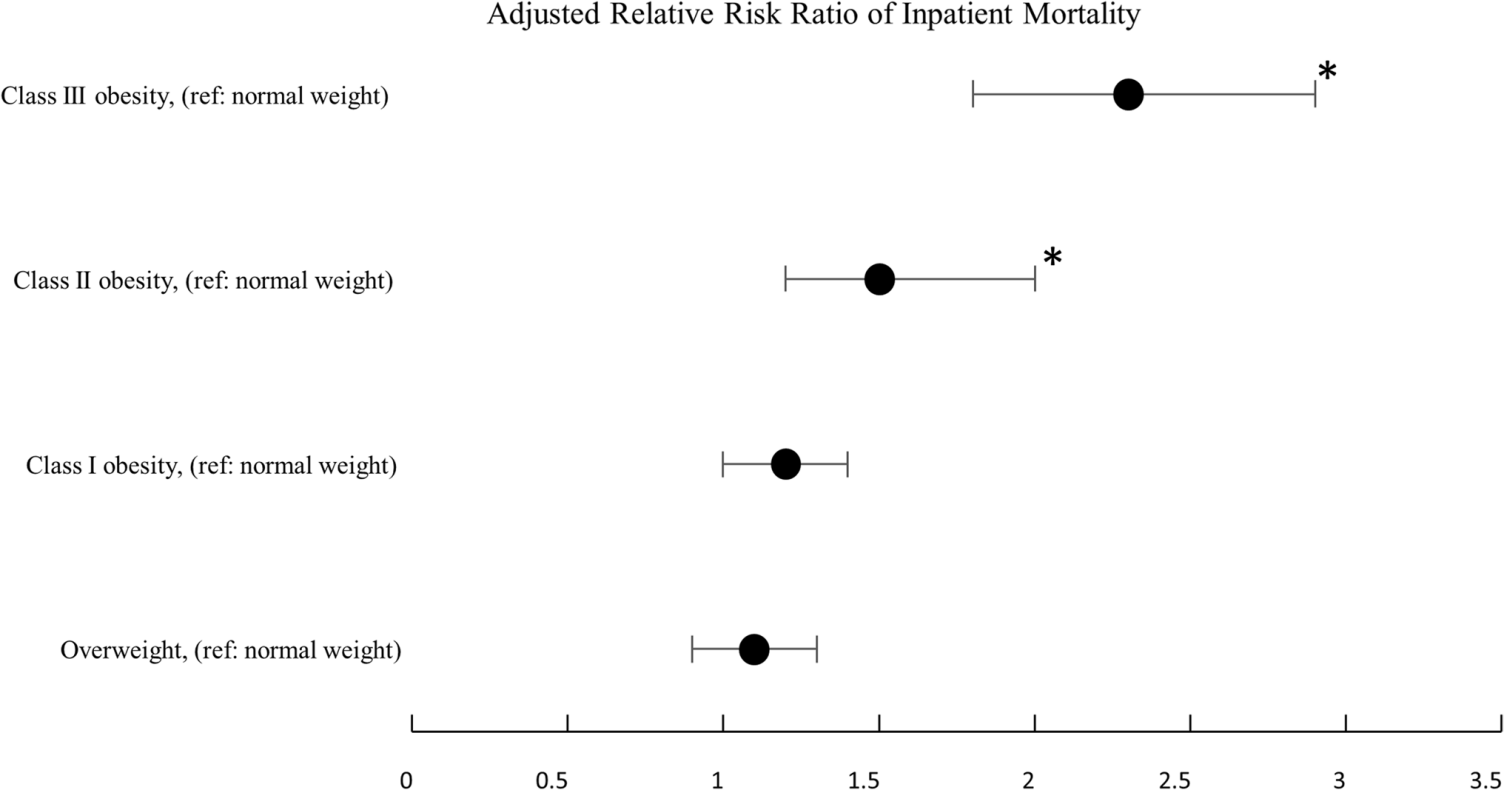
Adjusted relative risk ratio of inpatient mortality compared to normal weight by body mass index class (*denotes *p* < 0.0125)

The risk of mortality complications was significantly associated with the WHO obesity class: class II obesity aRR = 1.5 (95% CI 1.2–2.0, *p* = 0.001) and class III obesity aRR = 2.3 (95% CI 1.8–2.9, *p* < 0.001) patients when compared to normal-weight patients (Fig. 5; Supplementary Table S7).

A progressive inclusion of complication subclasses with increasing obesity severity was demonstrated. With reference to normal weight, class I obesity patients had an increased adjusted risk of AKI and pulmonary complications, class II obesity conferred additional risk of cardiac complications, and class III obesity conferred the further addition of significantly greater surgical site infection and VTE risk (Table 3).

**Table 3:** Adjusted relative risk ratio of complication subclass by body mass index

BMI class	Acute kidney injury	Cardiac complication	Pulmonary complication	Surgical site infection	VTE
Overweight (ref: normal weight); aRR (95% CI)	1.2 (1.0, 1.4)	1.1 (0.9, 1.2)	1.1 (1.0, 1.1)	1.0 (0.9, 1.1)	1.0 (1.0, 1.1)
Class I obesity (ref: normal weight); aRR (95% CI)	**1.4 (1.2, 1.6)**	1.0 (0.9, 1.2)	**1.3 (1.2, 1.4)**	1.0 (0.8, 1.1)	1.0 (1.0, 1.1)
Class II obesity (ref: normal weight); aRR (95% CI)	**1.7 (1.4, 2.0)**	**1.5 (1.2, 1.7)**	**1.4 (1.3, 1.6)**	1.1 (0.9, 1.3)	1.1 (1.0, 1.2)
Class III obesity (ref: normal weight); aRR (95% CI)	**2.3 (1.9, 2.9)**	**1.4 (1.1, 1.7)**	**1.8 (1.7, 2.0)**	**1.3 (1.1, 1.6)**	**1.2 (1.0, 1.3)**

aRR, Adjusted Relative Risk Ratio; BMI, Body Mass Index; CI, Confidence Interval; ref, Reference Variable; and VTE, Venous Thromboembolism. Bold = *p* < 0.0125

## Discussion

The present study aimed to identify the effects of obesity on short-term complications in patients with acetabular fractures. Using a large national database and rigorously adjusting for potential confounding as well as multiple hypothesis testing, we identified that increasing severity of obesity as classified by the World Health Organization is associated with 10–30% greater risk of inpatient complications, including serious adverse events, infections, intensive care unit needs, need for prolonged mechanical ventilation, as well as a 130–140% increased risk of mortality. In particular, class III obese patients faced greater incremental risk of preventable complications including surgical site infection and venous thromboembolism. To our knowledge, this is a novel report on short-term complications in both operatively and non-operatively treated acetabular fracture patients stratified over the full range of obesity classification.

About 20% of patients who sustain an acetabular fracture experience complications during the index admission, with in-hospital mortality rates ranging from 1.3 to 4.0% [6, 14, 15]. Our findings are consistent with several prior reports of increased risk of complications in obese patients. Carson et al. [16] compared 1331 morbidly obese patients to 45,119 non-morbidly obese patients and found a twofold greater risk of any complications in the morbidly obese group across both non-operative and operative treatment modalities. Using the same stratification, Porter et al. [15] also reported significantly increased rates of complications in morbidly obese patients when compared to non-morbidly obese patients. Similar results were found in smaller studies by Morris et al. and Karunakar et al. [10, 17] who treated BMI as a continuous variable and found significant associations between complication rates and increasing BMI. The mechanism of this effect is undoubtedly multifactorial, ranging from increased mechanical forces mitigating injury severity, a pro-inflammatory baseline state affecting the response to trauma, reduced cardiopulmonary reserve, and insulin resistance impacting bone and soft tissue healing [18, 19].

Our findings contrast with the “obesity paradox” of greater survival observed in obese hip fracture patients, as well as studies suggesting no increased risk of complications in obese patients undergoing acetabular fracture repair [11, 12]. Amin et al. [20] identified the probable contribution of survival bias to the “obesity paradox” observed in hip fracture patients and reported that only class III obese patients over the age of 75 years had decreased 30-day mortality after low-energy hip fracture. In a recent retrospective study of 428 operatively treated acetabular fractures, Lameka et al. [11] found no association between morbid obesity and inpatient complications, including ARDS, sepsis, pneumonia, VTE, or death. Our contrasting findings likely reflect the statistical power of a larger cohort selected from a database designed for the assessment of severely injured patients with rigorous adjustment for confounding and strict stratification of outcomes across obesity classes.

We did not observe differences in complication rates or mortality by operative versus non-operative treatment after adjustment for obesity class, injury severity, age, and comorbid conditions. While many acetabular fractures require treatment, obesity may influence the decision to perform a surgical repair and thus introduce indication bias in the assessment of risk. Morris et al. and Carson et al. [16, 17] also reported no differences between operative and non-operative treatments. However, delays in operative fixation of > 5 days were significantly associated with increased risk of complications, including SAE and infectious complications. Similar results have been reported in prior works, thus reinforcing the notion that delayed surgery is associated with greater complication rates [21]. There was no adjusted risk of mortality associated with treatment method or timing of operative fixation. In operatively treated patients, the rate of surgical site infection was 4.4% which is similar to the previous reports that demonstrated surgical site infection rates between 2.3 and 7.6% [22, 23]. Importantly, we found that the risk of surgical site infection was significantly increased only in class III obesity patients after adjusting for confounding.

The risk of clinically relevant VTE requiring medical intervention was increased only in patients with class III obesity after adjusting for confounding factors, including the mode of VTE chemoprophylaxis. Previously, Shaath et al. [9] reported a significantly increased risk of VTE in patients with BMI > 40 kg/m^2^ in their cohort of 333 operatively treated acetabular fractures. These results contrast with the studies by Lemeka et al. and Porter et al. [11, 15] who showed no such risk in their cohorts of obese acetabular fracture patients. Our data may support clinical investigation of the treatment effect of aggressive VTE prophylaxis such as IVC filter use or increased dosing of chemoprophylaxis in class III obese patients with acetabular fractures.

Our study is not without limitations. The use of a large database is inherently at risk for errors, including those related to data reporting and processing, as well as limitations in follow-up and generalizability. The risks of individual complications, especially late events, are likely underreported, as these data are limited to the initial admission. However, many serious systemic complications associated with severe trauma occur within the first 1–2 weeks after admission, a period we observed for the majority of patients [24]. The timing or specific etiology of complications and mortality are not available in the TQIP database, thus limiting the specificity of the reported outcomes. The high rate of non-operative treatment in this cohort should also be noted and likely reflects the inclusion of an elderly population with low-energy fractures who are able to be mobilized without operative intervention. Additionally, patients who were stabilized and transferred, as well as those who died before orthopedic intervention, would not be captured by the database. While this is a potential confounding factor, our findings remained significant when accounting for the mode of treatment; thus, BMI confers an increased risk of morbidity regardless of treatment method. We must also acknowledge that obesity classification by BMI may not be the most effective marker for overall health and may inaccurately classify patients with greater muscle mass or altered fat distribution. However, BMI remains the most commonly used proxy for body habitus in obese patients, thus remaining a relevant predictive marker in the clinical setting.

## Conclusion

Obesity is a growing pandemic condition independently associated with a severity-dependent risk of complications and mortality during the index hospitalization for an acetabular fracture. Class III obese patients face a greater incremental risk of preventable complications, including surgical site infection and venous thromboembolism. Clinical investigation of interventions to modify the risk of adverse events in this subgroup of acetabular fracture patients may be indicated.

## Supplementary Information

Below is the link to the electronic supplementary material.

Supplementary file1 (DOCX 57 KB)

## Data Availability

Data analyzed in the present study are available online through the Trauma Quality Improvement Program at https://www.facs.org/quality-programs/trauma/quality/trauma-quality-improvement-program/.
