# Supplementary material for: Obesity classification predicts early complications and mortality after acetabular fracture
Source: Eur J Orthop Surg Traumatol. 2023 Jul 6;34(7):3473–81. doi: 10.1007/s00590-023-03633-8 (PMC11490521; doi:10.1007/s00590-023-03633-8)
Supplement: Supplementary file 1 — Supplementary file1 (DOCX 57 KB) [file 590_2023_3633_MOESM1_ESM.docx]

Supplementary Table S1. 2005 Abbreviated Injury Scale and International Statistical Classification of Diseases and Related Health Problems 10^th^ coding.

| 2005 AIS acetabular fracture | "8562..” |
| --- | --- |
| One Column | "856251" "856252" |
| Transverse | "856261" "856262" |
| Associated Both Column | "856271" "856272" |
| 2005 AIS pelvis injury | "8561.." |
| 2005 AIS femur fracture | “8530..” “8531..” “8532..” “8533..” |
| 2005 AIS tibia fracture | “8540..” “8541..” “8542..” “8543..” |
| ICD10-PCS acetabular ORIF | “0QS404Z” “0QS504Z” “0QH404Z” “0QH504Z” “0QQ40ZZ” “0QQ50ZZ” |
| ICD10-PCS acetabular CRIF | “0QS434Z” “0QS444Z” “0QS534Z” “0QS544Z” “0QH434Z” “0QH444Z” “0QH534Z” “0QH544Z” “0QQ43ZZ” “0QQ44ZZ” “0QQ53ZZ” “0QQ54ZZ” |
| Abbreviations: AIS=Abbreviated Injury Scale, CRIF=Closed Reduction Internal Fixation, ICD10=International Statistical Classification of Diseases and Related Health Problems 10th coding, ORIF=Open Reduction Internal Fixation, PCS=Procedural Coding System | |

Supplementary Table S2. Comprehensive list of covariates tested during model selection.

| All Tested Covariates | Age, sex, body mass index, all race/ethnicity covariates (American Indian, Asian, Black, Hispanic, White), all comorbid conditions (alcoholism, angina, anticoagulant use, bleeding disorders, congestive heart failure, chronic obstruction pulmonary disease, dementia, diabetes, end stage renal disease, functionally dependent health status, history of myocardial infarction, history of stroke, hypertension, mental disorder, peripheral artery disease, smoking, steroid use, and substance abuse), vitals on admission (pulse rate, respiratory rate, oxygen saturation, temperature, systolic blood pressure (SBP)), lowest SBP, Glasgow Coma Score, Injury Severity Score, AIS anatomic region severity scores 1-8, acetabular fracture type (one column, transverse, associated both column, or open fracture), associated pelvic ring injury, concomitant lower extremity injury (femoral fracture, tibial fracture), minutes spent in the emergency department before admission, admitting facility trauma level (I-III),  teaching status, total bed number (≤200, 201-400, 401-600, ≥601), not-for-profit status, interfacility transfer to treating hospital, method of definitive operative fixation of acetabular fracture (nonoperative, closed reduction internal fixation (CRIF), open reduction internal fixation (ORIF)), time to acetabular fixation, treatment by exploratory laparotomy of the peritoneal cavity, pelvic angioembolization, volume of packed red blood cells, plasma, platelets, and cryoprecipitate given during the first 4 and 24 hours of admission, and venous thromboembolism (VTE) prophylaxis type (direct thrombin inhibitor, factor Xa inhibitor, heparin, low molecular weight heparin (LMHW), warfarin, other, none). |
| --- | --- |

Supplementary Table S3: Rate of inpatient complications and length of stay by body mass index class and method of treatment.

| **Total cohort** | **Normal weight** | **Overweight** | **Class I obesity** | **Class II obesity** | **Class III obesity** |
| --- | --- | --- | --- | --- | --- |
| Any inpatient complication, yes (%) | 11,818 (35.4) | 11,932 (35.9) | 6,744 (37.3) | 3,066 (38.8) | 2,881 (41.3) |
| SAE, yes (%) | 11,155 (33.4) | 11,593 (34.8) | 6,688 (36.9) | 3,011 (38.1) | 2,796 (40.0) |
| Any infectious complication, yes (%) | 3,735 (11.2) | 3,661 (11.0) | 2,195 (12.1) | 992 (12.6) | 936 (13.4) |
| Death, yes | 998 (3.0) | 1,138 (3.4) | 616 (3.4) | 284 (3.6) | 280 (4.0) |
| Total hospital LOS, days (%) | 9.2 (9.1, 9.3) | 9.8 (9.7, 9.9) | 10.7 (10.5, 10.9) | 11.6 (11.2, 11.9) | 12.8 (12.5, 13.1) |
| ICU LOS, days (95% CI) | 6.9 (6.8, 7.1) | 7.2 (7.0, 7.3) | 8.2 (7.9, 8.3) | 8.6 (8.2, 8.9) | 9.3 (8.9, 9.7) |
| Days requiring ventilator (95% CI) | 7.3 (7.1, 7.6) | 7.4 (7.2, 7.6) | 8.3 (8.0, 8.6) | 8.3 (7.8, 8.7) | 9.2 (8.7, 9.6) |
| **Non-operative treatment** | **Normal weight** | **Overweight** | **Class I obesity** | **Class II obesity** | **Class III obesity** |
| Any inpatient complication, yes (%) | 9,006 (34.6) | 8,586 (35.1) | 4,515 (36.3) | 1,964 (38.6) | 1,755 (39.9) |
| SAE, yes (%) | 8,411 (32.3) | 8,322 (34.0) | 4,494 (36.2) | 1,904 (37.5) | 1,700 (38.7) |
| Any infectious complication, yes (%) | 2,858 (11.0) | 2,632 (10.8) | 1,465 (11.8) | 597 (11.7) | 573 (13.0) |
| Death, yes | 937 (3.6) | 1,061 (4.3) | 572 (4.6) | 254 (5.0) | 243 (5.5) |
| Total hospital LOS, days (%) | 8.5 (8.4, 8.7) | 9.1 (8.9, 9.2) | 9.8 (9.6, 10.0) | 11.0 (10.6, 11.4) | 12.1 (11.6, 12.5) |
| ICU LOS, days (95% CI) | 6.8 (6.6, 7.0) | 7.2 (7.0, 7.4) | 8.2 (8.0, 8.5) | 8.8 (8.3, 9.2) | 9.5 (9.0, 9.9) |
| Days requiring ventilator (95% CI) | 7.5 (7.2, 7.8) | 7.6 (7.3, 7.8) | 8.5 (8.1, 8.9) | 8.6 (8.0, 9.2) | 9.8 (9.2, 10.5) |
| **Operative treatment** | **Normal weight** | **Overweight** | **Class I obesity** | **Class II obesity** | **Class III obesity** |
| Any inpatient complication, yes (%) | 2,812 (38.3) | 3,346 (37.9) | 2,250 (39.6) | 1,162 (41.2) | 1,126 (43.5) |
| SAE, yes (%) | 2,744 (37.3) | 3,271 (37.1) | 2,194 (38.7) | 1,107 (39.3) | 1,096 (42.4) |
| Any infectious complication, yes (%) | 877 (11.9) | 1,029 (11.7) | 730 (12.9) | 395 (14.0) | 363 (14.0) |
| Death, yes | 61 (0.8) | 77 (0.9) | 44 (0.8) | 30 (1.1) | 37 (1.4) |
| Total hospital LOS, days (%) | 11.5 (12.3, 11.7) | 11.8 (11.5, 12.0) | 12.6 (12.3, 12.9) | 12.6 (12.2, 13.0) | 14.1 (13.7, 14.6) |
| ICU LOS, days (95% CI) | 7.3 (7.0, 7.6) | 7.1 (6.9, 7.4) | 8.0 (7.6, 8.6) | 8.2 (7.8, 8.7) | 9.1 (8.5, 9.6) |
| Days requiring ventilator (95% CI) | 6.9 (6.4, 7.3) | 7.1 (6.7, 7.4) | 7.7 (7.2, 8.3) | 7.6 (7.0, 8.2) | 8.0 (7.3, 8.7) |

Supplementary Table S4. Multiple Logistic Regression on risk of any inpatient complication.

| **Primary covariate** | **Adjusted relative risk** | **95% CI** | **p-value** |
| --- | --- | --- | --- |
| BMI |  |  |  |
| Overweight, (ref: normal weight) | 1.0 | 1.0, 1.1 | 0.930 |
| Class I obesity, (ref: normal weight) | 1.1 | 1.0, 1.2 | 0.012 |
| Class II obesity, (ref: normal weight) | 1.2 | 1.1, 1.3 | <0.001 |
| Class III obesity, (ref: normal weight) | 1.3 | 1.2, 1.4 | <0.001 |
| **Selected model covariates** |  |  |  |
| Age | 1.0 | 1.0, 1.0 | <0.001 |
| Sex | 1.1 | 1.0, 1.1 | <0.001 |
| Alcoholism | 1.1 | 1.0, 1.2 | 0.003 |
| Bleeding disorder | 1.2 | 1.0, 1.3 | 0.007 |
| COPD | 1.3 | 1.1, 1.4 | <0.001 |
| Diabetes | 1.4 | 1.3, 1.5 | <0.001 |
| Mental disorder | 1.1 | 1.0, 1.2 | 0.001 |
| Hypertension | 1.1 | 1.1, 1.2 | <0.001 |
| Pulse rate | 1.0 | 1.0, 1.0 | <0.001 |
| GCS | 1.0 | 1.0, 1.0 | <0.001 |
| ISS | 1.0 | 1.0, 1.0 | <0.001 |
| AIS Region 4, Thorax | 1.0 | 1.0, 1.1 | <0.001 |
| AIS Region 5, Abdomen | 1.0 | 1.0, 1.1 | 0.001 |
| AIS Region 6, Spine | 1.1 | 1.0, 1.1 | 0.001 |
| Acetabular fracture type |  |  |  |
| Transverse, (ref one column) | 1.1 | 1.0, 1.0 | 0.038 |
| Associated Both Column, (ref one column) | 1.1 | 1.0, 1.2 | 0.002 |
| Concomitant injury, pelvis | 1.1 | 1.0, 1.2 | <0.001 |
| Hospital bed size |  |  |  |
| <200, (ref >600) | 1.0 | 1.0, 1.2 | 0.344 |
| 200-400, (ref >600) | 1.1 | 1.0, 1.2 | <0.001 |
| 401-600, (ref >600) | 1.2 | 1.1, 1.2 | <0.001 |
| Teaching status | 0.9 | 0.9, 0.9 | <0.001 |
| Time to acetabular fixation |  |  |  |
| <1 day, (ref 1-3 days) | 1.0 | 0.9, 1.1 | 0.894 |
| 3.1-5 days, (ref 1-3 days) | 1.1 | 1.0, 1.2 | 0.189 |
| >5 days, (ref 1-3 days) | 1.6 | 1.4, 1.7 | <0.001 |
| Exploratory laparotomy | 1.5 | 1.3, 1.7 | <0.001 |
| Units pRBCs given within 24 hours of admission |  |  |  |
| <1 unit, (ref none) | 0.9 | 0.64, 1.3 | 0.559 |
| 1-4 units, (ref none) | 1.2 | 1.1, 1.3 | 0.001 |
| 4.1-8 units, (ref none) | 1.8 | 1.6, 2.0 | <0.001 |
| 8.1-12 units, (ref none) | 2.0 | 1.7, 2.3 | <0.001 |
| >12 units, (ref none) | 2.8 | 2.3, 3.3 | <0.001 |
| VTE prophylaxis type |  |  |  |
| Direct thrombin inhibitor, (ref LMWH) | 0.9 | 0.4, 1.8 | 0.712 |
| Heparin, (ref LMWH) | 1.3 | 1.2, 1.3 | <0.001 |
| None, (ref LMWH) | 0.8 | 0.7, 0.9 | <0.001 |
| Xa inhibitor, (ref LMWH) | 1.0 | 0.6, 1.1 | 0.791 |
| Warfarin, (ref LMWH) | 0.8 | 0.6, 1.1 | 0.274 |
| Other, (ref LMWH) | 0.9 | 0.8, 1.0 | 0.020 |
| Abbreviations: AIS=Abbreviated Injury Score, BMI=Body Mass Index, COPD=Chronic Obstructive Pulmonary Disease, GCS=Glasgow Coma Scale, ISS=Injury Severity Score, LMWH=Low Molecular Weight Heparin, ORIF=Open Reduction Internal Fixation, pRBC=Packed Red Blood Cells, ref=Reference Variable, VTE=Venous Thromboembolism | | | |

Supplementary Table S5. Multiple Logistic Regression on risk of serious adverse event.

| **Primary covariate** | **Adjusted relative risk** | **95% CI** | **p-value** |
| --- | --- | --- | --- |
| BMI |  |  |  |
| Overweight, (ref: normal weight) | 1.0 | 1.0, 1.1 | 0.847 |
| Class I obesity, (ref: normal weight) | 1.1 | 1.0, 1.2 | 0.005 |
| Class II obesity, (ref: normal weight) | 1.2 | 1.1, 1.3 | <0.001 |
| Class III obesity, (ref: normal weight) | 1.4 | 1.3, 1.5 | <0.001 |
| **Selected model covariates** |  |  |  |
| Age | 1.0 | 1.0, 1.0 | <0.001 |
| Sex | 1.1 | 1.1, 1.2 | <0.001 |
| Alcoholism | 1.1 | 1.1, 1.2 | 0.002 |
| Cirrhosis | 1.3 | 1.1, 1.5 | <0.001 |
| COPD | 1.4 | 1.2, 1.5 | <0.001 |
| Diabetes | 1.7 | 1.6, 1.8 | <0.001 |
| Mental disorder | 1.1 | 1.1, 1.2 | 0.001 |
| Pulse | 1.0 | 1.0, 1.0 | <0.001 |
| GCS | 0.8 | 0.8, 0.8 | <0.001 |
| ISS | 1.0 | 1.0, 1.0 | <0.001 |
| AIS Region 1, Head | 1.0 | 1.0, 1.0 | <0.001 |
| AIS Region 2, Face | 1.1 | 1.1, 1.1 | <0.001 |
| AIS Region 3, Neck | 1.1 | 1.1, 1.2 | <0.001 |
| AIS Region 4, Thorax | 1.1 | 1.0, 1.1 | <0.001 |
| AIS Region 5, Abdomen | 1.0 | 1.0, 1.1 | 0.009 |
| AIS Region 6, Spine | 1.1 | 1.0, 1.2 | 0.002 |
| AIS Region 7, Upper Extremity | 1.0 | 1.0, 1.1 | <0.001 |
| Acetabular fracture type |  |  |  |
| Transverse, (ref one column) | 1.0 | 1.0, 1.1 | 0.440 |
| Associated Both Column, (ref one column) | 1.1 | 1.0, 1.1 | 0.012 |
| Concomitant injury, femur | 1.1 | 1.1, 1.2 | <0.001 |
| Concomitant injury, tibia | 1.2 | 1.1, 1.3 | <0.001 |
| Hospital bed size |  |  |  |
| <200, (ref >600) | 1.1 | 1.0, 1.2 | 0.011 |
| 200-400, (ref >600) | 1.1 | 1.1, 1.1 | 0.012 |
| 401-600, (ref >600) | 1.0 | 1.0, 1.1 | 0.416 |
| Interfacility transfer | 0.9 | 0.8, 0.9 | <0.001 |
| Time to acetabular fixation |  |  |  |
| <1 day, (ref 1-3 days) | 1.0 | 1.0, 1.1 | 0.367 |
| 3.1-5 days, (ref 1-3 days) | 1.2 | 1.1, 1.3 | 0.001 |
| >5 days, (ref 1-3 days) | 1.8 | 1.6, 2.0 | <0.001 |
| Exploratory laparotomy | 2.7 | 2.3, 3.1 | <0.001 |
| Units pRBCs given within 24 hours of admission |  |  |  |
| <1 unit, (ref none) | 1.7 | 1.2, 2.4 | 0.002 |
| 1-4 units, (ref none) | 1.9 | 1.7, 2.1 | <0.001 |
| 4.1-8 units, (ref none) | 4.5 | 3.9, 5.2 | <0.001 |
| 8.1-12 units, (ref none) | 10.5 | 7.8, 14.1 | <0.001 |
| >12 units, (ref none) | 24.0 | 15.1, 38.1 | <0.001 |
| VTE prophylaxis type |  |  |  |
| Direct thrombin inhibitor, (ref LMWH) | 1.5 | 0.7, 2.9 | 0.272 |
| Heparin, (ref LMWH) | 1.4 | 1.3, 1.5 | <0.001 |
| None, (ref LMWH) | 0.8 | 0.7, 0.9 | 0.001 |
| Other, (ref LMWH) | 1.0 | 0.9, 1.2 | 0.446 |
| Xa inhibitor, (ref LMWH) | 1.1 | 0.9, 1.4 | 0.482 |
| Warfarin, (ref LMWH) | 1.1 | 0.8, 1.6 | 0.434 |
| Abbreviations: AIS=Abbreviated Injury Score, BMI=Body Mass Index, COPD=Chronic Obstructive Pulmonary Disease, GCS=Glasgow Coma Scale, ISS=Injury Severity Score, LMWH=Low Molecular Weight Heparin, pRBC=Packed Red Blood Cells, ref=Reference Variable, VTE=Venous Thromboembolism | | | |

Supplementary Table S6. Multiple Logistic Regression on risk of infectious complications.

| **Primary covariate** | **Adjusted relative risk** | **95% CI** | **p-value** |
| --- | --- | --- | --- |
| BMI |  |  |  |
| Overweight, (ref: normal weight) | 1.0 | 0.9, 1.1 | 0.842 |
| Class I obesity, (ref: normal weight) | 1.1 | 1.0, 1.2 | 0.071 |
| Class II obesity, (ref: normal weight) | 1.2 | 1.1, 1.3 | 0.005 |
| Class III obesity, (ref: normal weight) | 1.3 | 1.2, 1.5 | <0.001 |
| **Selected model covariates** |  |  |  |
| Age | 1.0 | 1.0, 1.0 | <0.001 |
| Alcoholism | 1.1 | 1.1, 1.4 | <0.001 |
| Bleeding disorder | 1.3 | 1.1, 1.5 | <0.001 |
| CHF | 1.2 | 1.1, 1.3 | 0.008 |
| Cirrhosis | 1.4 | 1.2, 1.6 | <0.001 |
| COPD | 1.2 | 1.0, 1.3 | 0.0110 |
| Diabetes | 1.4 | 1.2, 1.5 | <0.001 |
| Mental disorder | 1.2 | 1.0, 1.3 | 0.006 |
| Smoking | 1.2 | 1.1, 1.3 | <0.001 |
| Substance abuse | 1.2 | 1.0, 1.3 | 0.010 |
| Pulse rate | 1.0 | 1.0, 1.0 | <0.001 |
| GCS | 1.0 | 1.0, 1.0 | <0.001 |
| ISS | 1.0 | 1.0, 1.0 | <0.001 |
| AIS Region 1, Head | 1.0 | 1.0, 1.1 | 0.008 |
| AIS Region 3, Neck | 1.1 | 1.0, 1.2 | 0.008 |
| AIS Region 5, Abdomen | 1.1 | 1.0, 1.1 | <0.001 |
| AIS Region 6, Spine | 1.1 | 1.0, 1.2 | 0.002 |
| Acetabular fracture type |  |  |  |
| Transverse, (ref one column) | 1.2 | 1.1, 1.2 | 0.001 |
| Associated Both Column, (ref one column) | 1.2 | 1.1, 1.3 | <0.001 |
| Time to acetabular fixation |  |  |  |
| <1 day, (ref 1-3 days) | 1.0 | 0.9, 1.1 | 0.840 |
| 3.1-5 days, (ref 1-3 days) | 1.0 | 0.9, 1.1 | 0.486 |
| >5 days, (ref 1-3 days) | 1.3 | 1.1, 1.5 | 0.001 |
| Exploratory laparotomy | 1.2 | 1.0, 1.4 | 0.011 |
| Units pRBCs given within 24 hours of admission |  |  |  |
| <1 unit, (ref none) | 0.9 | 0.6, 1.3 | 0.444 |
| 1-4 units, (ref none) | 1.1 | 1.0, 1.1 | 0.133 |
| 4.1-8 units, (ref none) | 1.8 | 1.5, 2.0 | <0.001 |
| 8.1-12 units, (ref none) | 2.6 | 2.1, 3.2 | <0.001 |
| >12 units, (ref none) | 3.2 | 2.6, 3.9 | <0.001 |
| VTE prophylaxis type |  |  |  |
| Direct thrombin inhibitor, (ref LMWH) | 0.7 | 0.3, 1.7 | 0.429 |
| Heparin, (ref LMWH) | 1.7 | 1.6, 1.9 | <0.001 |
| None, (ref LMWH) | 0.8 | 0.7, 0.9 | <0.001 |
| Other, (ref LMWH) | 0.8 | 0.7, 1.0 | 0.036 |
| Xa inhibitor, (ref LMWH) | 1.0 | 0.7, 1.3 | 0.751 |
| Warfarin, (ref LMWH) | 0.4 | 0.1, 1.1 | 0.066 |
| Abbreviations: AIS=Abbreviated Injury Score, BMI=Body Mass Index, CHF=Congestive Heart Failure, COPD=Chronic Obstructive Pulmonary Disease, GCS=Glasgow Coma Scale, ISS=Injury Severity Score, LMWH=Low Molecular Weight Heparin, pRBC=Packed Red Blood Cells, ref=Reference Variable, SBP=Systolic Blood Pressure, VTE=Venous Thromboembolism | | | |

Supplementary Table S7. Multiple Logistic Regression on risk of death.

| **Primary covariate** | **Adjusted relative risk** | **95% CI** | **p-value** |
| --- | --- | --- | --- |
| BMI |  |  |  |
| Overweight, (ref: normal weight) | 1.1 | 0.9, 1.3 | 0.208 |
| Class I obesity, (ref: normal weight) | 1.2 | 1.0, 1.4 | 0.120 |
| Class II obesity, (ref: normal weight) | 1.5 | 1.2, 2.0 | 0.001 |
| Class III obesity, (ref: normal weight) | 2.3 | 1.8, 2.9 | <0.001 |
| **Selected model covariates** |  |  |  |
| Age | 1.1 | 1.0, 1.1 | <0.001 |
| Sex | 1.6 | 1.4, 1.8 | <0.001 |
| Cirrhosis | 2.1 | 1.5, 2.8 | <0.001 |
| Diabetes | 1.3 | 1.1, 1.6 | 0.002 |
| ESRD | 1.9 | 1.4, 2.7 | <0.001 |
| Functional disability | 1.7 | 1.3, 2.2 | <0.001 |
| Dementia | 1.6 | 1.2, 2.2 | 0.002 |
| GCS | 0.9 | 0.9, 0.9 | <0.001 |
| ISS | 1.0 | 1.0, 1.0 | <0.001 |
| AIS Region 1, Head | 1.1 | 1.1, 1.1 | <0.001 |
| Acetabular fracture type |  |  |  |
| Transverse, (ref one column) | 1.4 | 1.2, 1.7 | <0.001 |
| Associated Both Column, (ref one column) | 1.4 | 1.2, 1.6 | <0.001 |
| Exploratory laparotomy | 1.7 | 1.4, 2.2 | <0.001 |
| Units pRBCs given within 24 hours of admission |  |  |  |
| <1 unit, (ref none) | 2.3 | 1.3, 4.1 | 0.006 |
| 1-4 units, (ref none) | 1.5 | 1.2, 1.8 | <0.001 |
| 4.1-8 units, (ref none) | 1.4 | 1.1, 1.8 | 0.003 |
| 8.1-12 units, (ref none) | 1.4 | 1.0, 2.0 | 0.043 |
| >12 units, (ref none) | 3.1 | 2.3, 4.0 | <0.001 |
| VTE prophylaxis type |  |  |  |
| Direct thrombin inhibitor, (ref LMWH) | 0.2 | 0.0, 3.9 | 0.276 |
| Heparin, (ref LMWH) | 1.5 | 1.3, 1.8 | <0.001 |
| None, (ref LMWH) | 7.2 | 5.6, 9.2 | <0.001 |
| Other, (ref LMWH) | 1.9 | 1.4, 2.4 | <0.001 |
| Other, (ref LMWH) | 2.0 | 1.5, 2.6 | <0.001 |
| Xa inhibitor, (ref LMWH) | 0.5 | 0.2, 1.6 | 0.245 |
| Warfarin, (ref LMWH) | 0.7 | 0.3, 2.0 | 0.559 |
| Abbreviations: AIS=Abbreviated Injury Score, BMI=Body Mass Index, CHF=Congestive Heart Failure, ESRD=End Stage Renal Disease, GCS=Glasgow Coma Scale, ISS=Injury Severity Score, LMWH=Low Molecular Weight Heparin, pRBC=Packed Red Blood Cells, ref=Reference Variable, VTE=Venous Thromboembolism | | | |
